# PERIOD Phosphoclusters Control Temperature Compensation of the *Drosophila* Circadian Clock

**DOI:** 10.3389/fphys.2022.888262

**Published:** 2022-06-02

**Authors:** Radhika Joshi, Yao D. Cai, Yongliang Xia, Joanna C. Chiu, Patrick Emery

**Affiliations:** ^1^ Department of Neurobiology, University of Massachusetts Chan Medical School, Worcester, MA, United States; ^2^ Department of Entomology and Nematology, University of California, Davis, Davis, CA, United States

**Keywords:** circadian rhythms, temperature compensation, period, phophorylated amino acids, drosophila

## Abstract

Ambient temperature varies constantly. However, the period of circadian pacemakers is remarkably stable over a wide-range of ecologically- and physiologically-relevant temperatures, even though the kinetics of most biochemical reactions accelerates as temperature rises. This thermal buffering phenomenon, called temperature compensation, is a critical feature of circadian rhythms, but how it is achieved remains elusive. Here, we uncovered the important role played by the *Drosophila* PERIOD (PER) phosphodegron in temperature compensation. This phosphorylation hotspot is crucial for PER proteasomal degradation and is the functional homolog of mammalian PER2 S478 phosphodegron, which also impacts temperature compensation. Using CRISPR-Cas9, we introduced a series of mutations that altered three Serines of the PER phosphodegron. While all three Serine to Alanine substitutions lengthened period at all temperatures tested, temperature compensation was differentially affected. S44A and S45A substitutions caused undercompensation, while S47A resulted in overcompensation. These results thus reveal unexpected functional heterogeneity of phosphodegron residues in thermal compensation. Furthermore, mutations impairing phosphorylation of the *per* ^*s*^ phosphocluster showed undercompensation, consistent with its inhibitory role on S47 phosphorylation. We observed that S47A substitution caused increased accumulation of hyper-phosphorylated PER at warmer temperatures. This finding was corroborated by cell culture assays in which S47A slowed down phosphorylation-dependent PER degradation at high temperatures, causing PER degradation to be excessively temperature-compensated. Thus, our results point to a novel role of the PER phosphodegron in temperature compensation through temperature-dependent modulation of the abundance of hyper-phosphorylated PER. Our work reveals interesting mechanistic convergences and differences between mammalian and *Drosophila* temperature compensation of the circadian clock.

## Introduction

Circadian clocks are present across almost all life forms and allow them to adapt their activities with daily changes in their surrounding environment. In eukaryotes, the molecular circadian pacemaker is a self-sustained transcriptional feedback loop that repeats itself every ∼24 h, thus producing molecular, physiological and behavioral rhythms that closely match the duration of the Earth’s revolution around its own axis (Dunlap, 1999). In *Drosophila*, the circadian clock comprises four core components (Hardin, 2011). The transcription factors CLOCK (CLK) and CYCLE (CYC) form a heterodimer that binds to E-box motifs upstream of the *period (per)* and *timeless (tim)* genes to promote their transcription. PER and TIM heterodimerize in the cytoplasm before entering the nucleus. Once inside the nucleus, the PER/TIM heterodimer binds to CLK/CYC and thus inhibits *per* and *tim* transcription. PER, TIM, and CLK undergo various post-translational modifications that affect the period of the clock, the most notable being the progressive phosphorylation driven by kinases such as DOUBLETIME (DBT) (Kloss et al., 1998; Price et al., 1998), SHAGGY (Martinek et al., 2001), Proline-directed kinases including p38 MAP Kinase (Ko et al., 2010; Dusik et al., 2014), CASEIN KINASE 1 alpha (CK1α) (Lam et al., 2018), CASEIN KINASE 2 (CK2) (Lin et al., 2002; Lin et al., 2005; Akten et al., 2003; Szabó et al., 2013), AMPK (Cho et al., 2019) and NEMO (NMO) (Chiu et al., 2011; Yu et al., 2011).

Circadian clocks exhibit three key properties (Pittendrigh, 1960). First, these clocks free-run with a period of ∼24 h in the absence of external temporal cues (referred to as Zeitgebers) such as the daily light and temperature cycles. Second, these Zeitgebers can reset the clocks, thus anchoring the clock’s phase to the day/night cycle. Third, the period of circadian clocks is temperature-compensated. As ambient temperature increases, most enzymatic reactions speed up. However, under constant conditions, circadian clocks exhibit ∼24 h periodicities over a wide range of physiological temperatures, even in ectotherms. This was first observed by C. Pittendrigh while studying eclosion rhythms of *Drosophila pseudoobscura* (Pittendrigh, 1954).

The mechanism of temperature compensation is one of the long lasting mysteries of circadian rhythms. Different models have been proposed. For example, the network model proposes that multiple temperature-sensitive reactions cancel each other with changes in temperature. Early studies on Dinoflagellates indeed suggested that two temperature-sensitive reactions with opposite effects would help achieving constant period (Hastings and Sweeney, 1957). At the biochemical level, phosphorylation mediated by CK1 and CK2 has received the most attention across species. Mutations in the β1 and α subunits of CK2 in *Neurospora* alter temperature compensation (Mehra et al., 2009). Temperature compensation in *Arabidopsis* is modulated by the inhibitory effect of CK2 phosphorylation on CIRCADIAN ASSOCIATED CLOCK A1 (CCA1) binding. CCA1 binding to its target promoters increases with temperature, which is kept in check by the opposing effect of CK2 phosphorylation (Portolé and Má, 2010). In mammals, phosphorylation of a PER2 peptide by CK1δ is temperature-insensitive, owing to opposite temperature sensitivity of substrate and product binding by this kinase (Isojima et al., 2009; Shinohara et al., 2017). Interestingly, the *CK1ε* ^*Tau*^ mutant shows an undercompensation phenotype (Tosini and Menaker, 1998; Zhou et al., 2015). Furthermore, CK1δ/ε activity on PER2 results in a temperature-dependent phosphoswitch involving two phosphorylation sites (Eide et al., 2005; Zhou et al., 2015; Masuda et al., 2020). One of them is Serine (S)-478, also called phosphodegron. S478 phosphorylation causes βTrCP (β-Transducing repeat Containing Protein)-dependent ubiquitination and degradation of mPER2 (Eide et al., 2005; Reischl et al., 2007; Masuda et al., 2020). The second is S659, the mouse homolog to the site of Familial Advanced Sleep Phase Syndrome (FASPS) mutations (Toh et al., 2001). S659 phosphorylation inhibits phosphorylation of the phosphodegron, and thus PER2 degradation (Shanware et al., 2011; Zhou et al., 2015). S478 is preferentially phosphorylated at colder temperatures while FASPS domain phosphorylation increases at warmer temperatures, consequently balancing out PER stability (Zhou et al., 2015; Narasimamurthy et al., 2018). Along with phosphorylation, other processes such as sumoylation in plants (Hansen et al., 2017), nuclear cytoplasmic ratio of PER (Giesecke et al., 2021) and TIM (Singh et al., 2019), PER intermolecular and PER-TIM interactions (Gekakis et al., 1995; Huang et al., 1993, Huang et al., 1995) have been proposed to modulate temperature compensation of circadian rhythms in flies.

**FIGURE 1:**
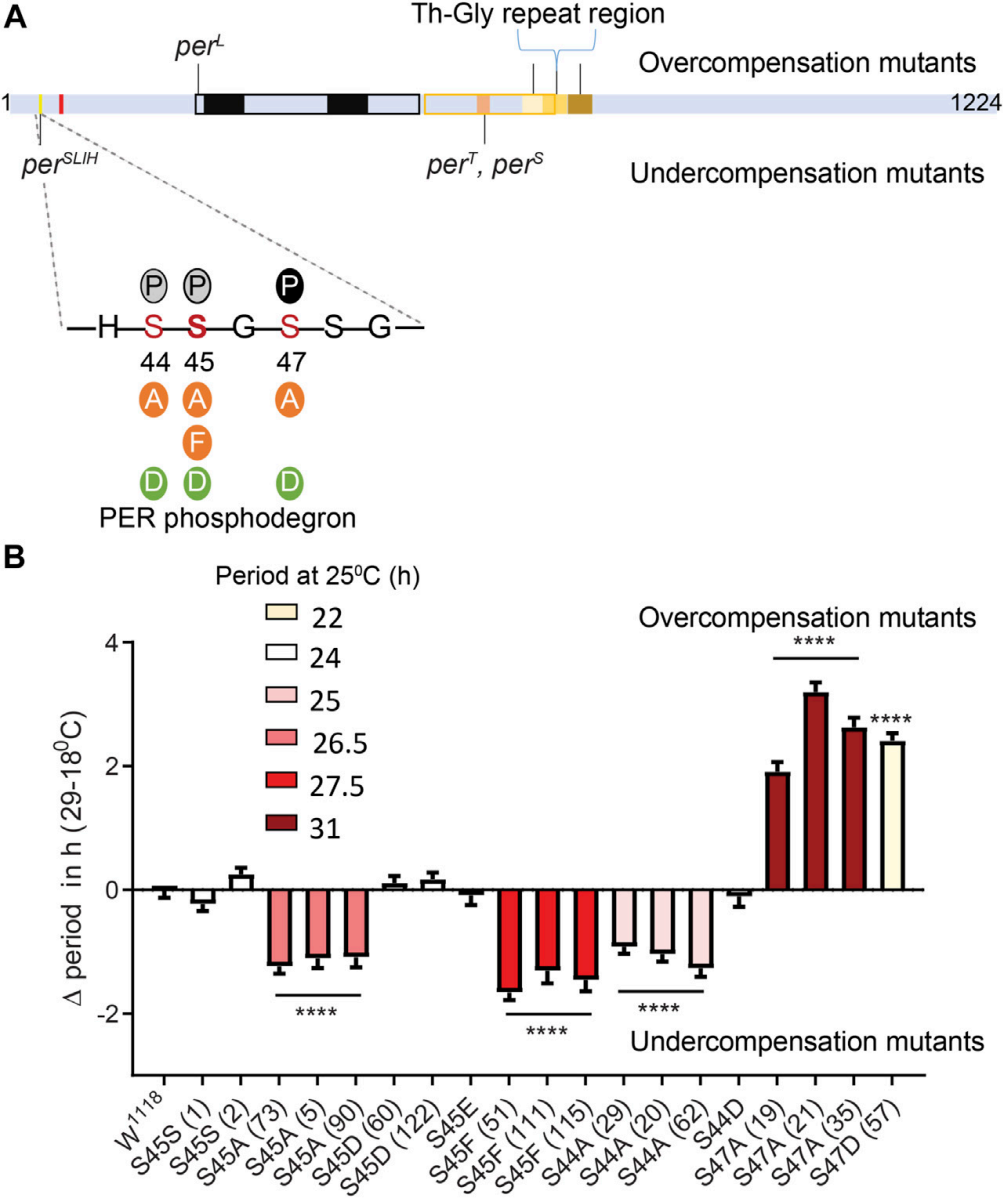
Differential modulation of temperature compensation by three Serine residues in the PER phosphodegron. **(A)** Schematic of PER protein depicting the relative positions of amino acids modified in various mutants affecting temperature compensation in *Drosophila*. Mutants above the PER schematic are overcompensated while mutants depicted below are undercompensated mutants. The magnified region of PER shows residues of the dPER phosphodegron and the various substitutions (Serine to Alanine, Phenylalanine, Aspartate or Glutamate) we generated using CRISPR/CAS9. Bold S at the 45th residue indicates the position of *per* ^*SLIH*^ mutation. Solid P within black circle, shows that S47 position undergoes phosphorylation. Grey circles indicate potential additional phosphorylation sites at S44 and S45 (Chiu et al., 2008). Red mark indicate a putative NLS close to the phosphodegron (Chang and Reppert, 2003). **(B)** Graphical representation of the difference in period values of various mutants of dPER phosphodegron at 29 and 18°C, relative to controls. ΔPeriod values for each genotype were offset by the average difference in period observed at 29 and 18°C in S45SI and S45SII control lines, so that overcompensation or undercompensation compared to control is clearly represented. The color scheme denotes period of each mutant line at 25°C. Interestingly, S44A, S45A, and S47A all produce long period phenotype at 25°C. However, the directionality of their temperature compensation phenotypes is opposite between S44A/S45A and S47A. S44A, and S45A produce significant undercompensation while S47A and S47D shows overcompensation. Statistical significance was calculated using two-way ANOVA with Sidak’s multiple comparison test.

Over the past several decades, circadian mutants have fuelled our understanding of the molecular clockwork. Therefore, to gain better understanding into the mechanism of temperature compensation in *Drosophila* we turned towards *Drosophila* mutants known to have a temperature compensation defect. Interestingly, of the numerous mutations impacting circadian rhythm period, only a subset of mutants show a defect in temperature compensation (Bao et al., 2001; Ewer et al., 1990; Hamblen et al., 1998; Konopka et al., 1994; Matsumoto et al., 1999; Peixoto et al., 1998; Rothenfluh et al., 2000; Sawyer et al., 1997). We were intrigued by the mutant *per* ^*SLIH*^ (*Some Like It Hot*, Figure 1A) due to its striking undercompensation phenotype: circadian period length decreases as temperature increases in this mutant instead of remaining approximately the same. (Hamblen et al., 1998). The *per^SLIH^* mutation causes a Serine (S) to Tyrosine (Y) substitution at the 45th residue, which is a part of *Drosophila* PER’s phosphodegron (S44, 45, and 47) (Chiu et al., 2008). The PER phosphodegron is regulated in a similar manner as its mammalian homolog (Chiu et al., 2011): it is phosphorylated by the CK1δ/ε homolog DBT (Kloss et al., 1998; Price et al., 1998; Chiu et al., 2008), and its phosphorylation is inhibited by the *per^short^ (per^s^)* phosphocluster, which is equivalent to the mammalian FASPS domain (Zhou et al., 2015). Phosphorylation events at the PER phosphodegron, S47 in particular, are critical for Supernumerary limbs (SLIMB)-mediated PER degradation and thus period length (Grima et al., 2002; Ko et al., 2002; Chiu et al., 2008). SLIMB is the fly homolog of βTrCP. Here, we show that, unexpectedly, the residues of the *Drosophila* phosphodegron are functionally heterogeneous and differentially modulate temperature compensation, even though they all lengthen circadian period. In addition, we found that the *per^s^* phosphocluster, which negatively regulates S47 phosphorylation, also regulates temperature compensation. Furthermore, we show that substituting PER’s S47 residue to an Alanine (A) results in an overcompensated circadian period, i.e., circadian period lengthens when ambient temperature increases. This overcompensation phenotype correlates with increased hyper-phosphorylated PER levels *in vivo* at warm temperature and overcompensated PER degradation in a well-established cell culture model. These results converge to support an important role of the phosphodegron in temperature compensation in *Drosophila*.

## Results

### Differential Impact of Three PER Phosphodegron Residues on Temperature Compensation

The unique location of the *per^SLIH^* mutation (Figure 1A) prompted us to evaluate the role of residues of the PER phosphodegron in temperature compensation. DBT, a Serine/Threonine kinase, phosphorylates S47, with S44 and S45 being additional potential targets (Chiu et al., 2008). To explore the possibility that phosphorylation at these residues modulates temperature compensation, we generated mutant *per* alleles encoding phosphomimics [S to D (Aspartate) or E (Glutamate)] and phosphoinhibitors [S to Alanine (A)] at PER S44, S45, and S47 residues using CRISPR-CAS9 (Figure 1A). Moreover, we generated a mutant *per* allele creating a S45F (S to Phenylalanine) substitution to mimic contribution of a bulky hydrophobic amino acid similar to Tyrosine (Y) to validate previous observations on *per^SLIH^*. We used S45S as a control, which contains only the silent substitutions introduced at the protospacer adjacent motifs (PAM site) during CRISPR mutagenesis. In our hands *white* ^*1118*^ (*w* ^*1118*^) and S45S control strains showed a decrease in period of ∼0.9 h (Table 1) over the range of 18–29°C. To accurately represent the effect of mutations on temperature compensation, values plotted on Figure 1B are relative to the average of the two S45S control strains, which was set to 0. Note that an undercompensation phenotype is represented with a minus value, since period shortens as temperature increases, while overcompensation is represented by a positive value.

**TABLE 1:** Circadian behavior of control and *per* mutant flies under different temperatures. Mutations in the phosphoclusters were generated by CRISPR/Cas9-mediated genome editing. Amino acid substitutions are indicated in the left column. *w* ^*1118*^ is a control strain. For the *per* ^*s*^ phosphocluster, *per* ^*0*^ flies were rescued with various transgenes. 13.2WT carries a wild-type *per* genomic region, while the other transgenes carry the indicated substitutions. AR = arrhythmic ND = Not done, N/A = Not applicable.

	Temperature°C	Period (h)	Period sem ±	% Rhythmic flies	# Rhythmic flies	Power	Power sem ±
PER phosphodegron							
*w* ^*1118*^	29	23.6	0.02	87.5	93	80	2.6
	25	24.5	0.05	86.9	53	76.1	3.5
	20	24.3	0.13	53.8	42	53.9	4
	18	24.5	0.09	40	32	64.1	5.5
S45S (1)	29	23.8	0.04	80.7	71	63.9	2.6
	25	25	0.06	95.7	44	67.4	3.6
	20	24.8	0.05	76.6	85	47.9	1.6
	18	25.1	0.08	76.6	36	66.6	6.1
S45S (2)	29	23.9	0.05	83.3	60	67.5	2.3
	25	24.9	0.04	95.3	61	74.9	3.4
	20	24.8	0.06	61	64	35.9	1.9
	18	24.7	0.07	84.7	50	63.7	4.5
S45F (51)	29	26.6	0.06	62.9	34	51.5	3.4
	25	27.7	0.07	83.9	26	70.5	6.2
	20	28.7	0.07	71.3	67	50	2.3
	18	29.2	0.06	87.1	54	64.7	3.5
S45F(111)	29	26.4	0.05	60	18	51.7	3.7
	25	27.3	0.21	53.3	8	56.8	12
	20	28.2	0.11	65.8	25	35.6	2.3
	18	28.7	0.16	37.5	12	40.5	5.6
S45F(115)	29	26.2	0.1	62.9	17	52.7	5
	25	27.5	0.11	90.9	20	76.3	6.8
	20	28.3	0.09	77.8	35	56.7	3
	18	28.7	0.16	67.7	21	57.2	4.9
S45A (73)	29	26.3	0.08	76.6	36	47.4	2.7
	25	27.2	0.09	93.5	29	83	5.9
	20	28	0.07	82.1	78	51.9	2
	18	28.5	0.06	87.5	56	70.1	4.4
S45A (5)	29	26	0.05	80.4	37	53.3	3.3
	25	26.7	0.09	62.5	10	53.2	4.3
	20	27.2	0.08	36.6	34	45.9	2.9
	18	28.1	0.14	60	18	45	5.4
S45A (90)	29	26.3	0.06	65	26	46	3.2
	25	26.8	0.13	93.8	15	87.5	10
	20	27.8	0.08	75.4	46	49	2.7
	18	28.4	0.12	70	21	69.5	6.7
S45D (67)	29	24.1	0.05	84.4	38	57.2	2.8
	25	24.9	0.04	100	32	84.1	4.6
	20	24.8	0.05	76	73	51.1	2
	18	24.9	0.04	95.1	58	80.7	4.1
S45D (122)	29	24	0.06	84.1	53	61.8	2.7
	25	24.9	0.04	95.1	58	76.4	3.2
	20	24.8	0.04	80	88	53.4	1.9
	18	24.8	0.05	91.1	51	70.1	4.2
S45E	29	23.9	0.04	87.2	41	59.9	3.6
	25	24.7	0.04	93.3	43	62.4	3.4
	20	24.2	0.08	44.2	19	37.9	2.8
	18	24.9	0.19	35.4	17	35.8	2.4
S44A (29)	29	24.9	0.04	88.6	70	63.1	2.4
	25	25.8	0.07	97.5	39	83.8	3.8
	20	25.7	0.1	82.1	69	53.1	2.2
	18	26.8	0.1	73.9	34	66.7	6.2
S44A (20)	29	24.9	0.06	77.1	37	64.1	3.1
	25	26	0.08	91.3	21	77.3	5.8
	20	26.6	0.06	84.8	56	55.7	2.9
	18	26.9	0.07	89.1	41	60.9	4.5
S44A (62)	29	24.7	0.17	76.7	33	49.9	3.4
	25	24.2	0.1	75	14	64.1	6.1
	20	ND	ND	ND	ND	ND	ND
	18	27	0.16	55	33	39.3	2.5
S44D	29	25.6	0.1	62.5	25	49.1	3.5
	25	26.6	0.05	90.7	39	63.3	2.8
	20	26.9	0.09	66.7	29	42.9	3.6
	18	26.7	0.13	35.7	20	35.9	2.8
S47A (19)	29	32.1	0.17	28.8	17	41.2	3.5
	25	31.3	0.1	53.8	44	43.5	2.5
	20	31	0.26	46.9	15	32.5	2.1
	18	31.2	0.14	52.1	49	51.1	3.5
S47A (21)	29	33.5	0.21	38.3	18	31.1	2
	25	31.5	0.11	44.9	48	56.3	4.3
	20	31.5	0.21	15.6	5	39.8	6.9
	18	31.3	0.15	58.8	47	60.1	4.5
S47A (35)	29	33	0.23	23.3	17	31.8	1.6
	25	31.5	0.07	72.9	70	48.7	2.5
	20	31.3	0.27	37.5	12	40.2	5.5
	18	31.4	0.13	47.7	53	44.5	3
S47D	29	22.5	0.03	88.5	46	75.4	3.6
	25	22.6	0.07	97.9	47	86.9	3.4
	20	21.7	0.05	93.6	44	54.6	3
	18	21.1	0.09	72.7	40	50.8	3.8
S4445A (105)	29	34.4	0.25	56.3	9	38.3	2.3
	25	35.9	0.2	32.3	10	52.5	5.9
	20	35.3	0.24	35.6	16	30.7	2.3
	18	38	0	6.3	1	22.7	0
S4445A (32)	29	34	0.11	19.4	6	36.4	5.9
	25	AR	N/A	0	0	N/A	N/A
	20	35.2	0.95	4.2	2	30.7	2.5
	18	AR	N/A	0	0	N/A	N/A
S4445A (60)	29	34.1	0.2	59.4	17	38.6	3.2
	25	AR	N/A	0	0	N/A	N/A
	20	35.6	0.5	25.4	16	40.6	4.4
	18	AR	N/A	0	0	N/A	N/A
S4547A (1)	29	AR	N/A	0	0	N/A	N/A
	25	AR	N/A	0	0	N/A	N/A
	20	AR	N/A	0	0	N/A	N/A
	18	AR	N/A	0	0	N/A	N/A
S4547A (30)	29	AR	N/A	0	0	N/A	N/A
	25	AR	N/A	0	0	N/A	N/A
	20	AR	N/A	0	0	N/A	N/A
	18	AR	N/A	0	0	N/A	N/A
S4547A (57)	29	AR	N/A	0	0	N/A	N/A
	25	AR	N/A	0	0	N/A	N/A
	20	AR	N/A	0	0	N/A	N/A
	18	AR	N/A	0	0	N/A	N/A
S4547A (84)	29	AR	N/A	0	0	N/A	N/A
	25	AR	N/A	0	0	N/A	N/A
	20	AR	N/A	0	0	N/A	N/A
	18	AR	N/A	0	0	N/A	N/A
S4547A (90)	29	AR	N/A	0	0	N/A	N/A
	25	AR	N/A	0	0	N/A	N/A
	20	AR	N/A	0	0	N/A	N/A
	18	AR	N/A	0	0	N/A	N/A
*per* ^*s*^ phosphocluster							
13.2WT	29	23.8	0.16	89.3	75	80.7	3.1
	25	24.2	0.06	76.6	36	66.9	6
	20	23.9	0.11	23.3	15	37.6	4.4
	18	23.8	0.09	32.9	27	50.4	4.1
S47A (M28-F1)	29	32.7	0.16	48.3	43	43.5	2.6
	25	30.5	0.16	44.7	21	47.5	3.6
	20	30	0.21	9.9	9	40.9	3.7
	18	29.9	0.13	27.2	28	33.8	2.3
S47A (F15-F1)	29	32.6	0.08	96.8	61	53.2	1.7
	25	31.6	0.07	95.8	46	77.8	6.4
	20	31.4	0.16	65.6	43	44.3	3
	18	29.8	0.14	50.8	32	52	3.8
S47D (M7-M2)	29	21.7	0.05	69.6	48	62	3.5
	25	21.8	0.06	80.4	37	63.6	4.2
	20	21.5	0.06	68.1	47	50.6	3
	18	21	0.09	44.8	23	51.7	5.5
S596A (F1-F1)	29	15.9	0.12	75	33	63.6	4.6
	25	15.2	0.04	91.5	43	86.1	4.1
	20	15.9	0.14	18.8	9	39.1	5.6
	18	16.3	0.17	8.5	4	31.4	4.3
S596A (M16-F1)	29	15.7	0.04	68.1	32	61.4	4
	25	15.5	0.03	93.8	45	113	5.7
	20	16.8	0.06	95.7	44	78.7	4
	18	17.2	0.27	81.6	62	87.6	4.4
TS583-596A (F30-M1)	29	16.9	0.17	22.9	11	33.3	5
	25	17.4	0.47	72.3	34	44.6	3.3
	20	17.7	0.09	60.4	29	44.7	2.9
	18	17.8	0.09	67.3	37	48.6	4.2
TS583-596A (M3-F1)	29	16.2	0.07	61.7	29	65.7	4.5
	25	16.3	0.07	80.9	38	80.4	5.7
	20	17	0.07	82.6	24	78.8	3.4
	18	17.3	0.22	86.8	79	94.3	4.8
TS583-596D (F2-F1)	29	15.3	0.06	55.3	26	60	4.7
	25	15	0.05	91.5	43	94.1	5.4
	20	15.8	0.08	45.8	22	55.2	3.9
	18	16	0.08	48.6	35	56.2	4.6
TS583-596D (F2-M1)	29	15.1	0.06	68.8	22	61.4	4
	25	15.2	0.07	78.6	22	72.1	6.4
	20	15.7	0.07	69	20	55.2	5.1
	18	16	0.05	60	37	48.7	3.8
S589A (M8-F1)	29	19.4	0.05	95.1	39	89.6	4.8
	25	19.6	0.04	97.9	46	127	5.1
	20	21	0.43	97.9	46	68.1	4.1
	18	20.7	0.08	76.7	56	76	5.1

Consistent with previous observations (Chiu et al., 2008), we observed that the phosphoinhibitory substitution S47A produced lengthened period. In addition, we found that S44A, S45A, and S45F also resulted in longer periods, albeit to a lesser extent at 25^°^C (Figure 1B; Table 1). S44A lengthened the period by ∼1.5 h, S45A by 2.5 h, and S47A by 6.5 h at 25°C. Interestingly, the effect of these mutations on temperature compensation did not correlate with period length. S44A and S45 A/F were undercompensated by ∼1–2 h, compared to controls. However, S47A showed an opposite phenotype: it caused a strong overcompensation of more than 2 h (Figure 1B).

Since it is possible that mutations at a residue affect phosphorylation events at the neighboring residues, we generated the double mutants S44-45A and S45-47A. S44-45A double mutants exhibited a very long period (>34 h), but the effect on temperature compensation was difficult to interpret due to poor rhythmicity, particularly at 18°C (Table 1). However, the synergistic effects on period and rhythmicity indicate that S44 and 45 work together to control circadian rhythms. S45–47A mutants were completely arrhythmic at both temperatures tested (Table 1).

We also tested phosphomimic substitutions (S to D/E). S45D and S45E did not show any substantial effect on either period length or temperature compensation (Figure 1; Table 1). S44D lengthened the period to 26.6 h at 25°C, but had no effect on temperature compensation. The behavior of S47D was the most peculiar. It showed a short rhythm period phenotype (>21 h), as previously reported by Chiu et al. (2008) with *per* genomic rescue constructs in an amorphic *per* ^*0*^ background (Konopka and Benzer, 1971). However, period was overcompensated by more than 2 h. This could either be because phosphomimics do not correctly or fully reproduce the structural changes caused by phosphorylation, or because phosphomimics are neither temporally controlled nor reversible and could thus have unexpected impact on temperature compensation.

Thus, taken together our results show that the PER phosphodegron modulates temperature compensation, and shows functional temperature compensation heterogeneity within its residues.

### Role of the *per* ^*s*^ Domain in Temperature Compensation

To further strengthen evidence for a role of the PER phosphodegron in temperature compensation, we analyzed the impact of mutations in the *per* ^*s*^ domain (T/S583-596). Indeed, this region also undergoes phosphorylation by DBT and NMO, and phosphorylation in the *per* ^*s*^ domain temporally precedes and inhibits phosphorylation at S47 (Chiu et al., 2011). Therefore we tested point mutations in the *per* ^*s*^ domain for their role in temperature compensation.

**FIGURE 2:**
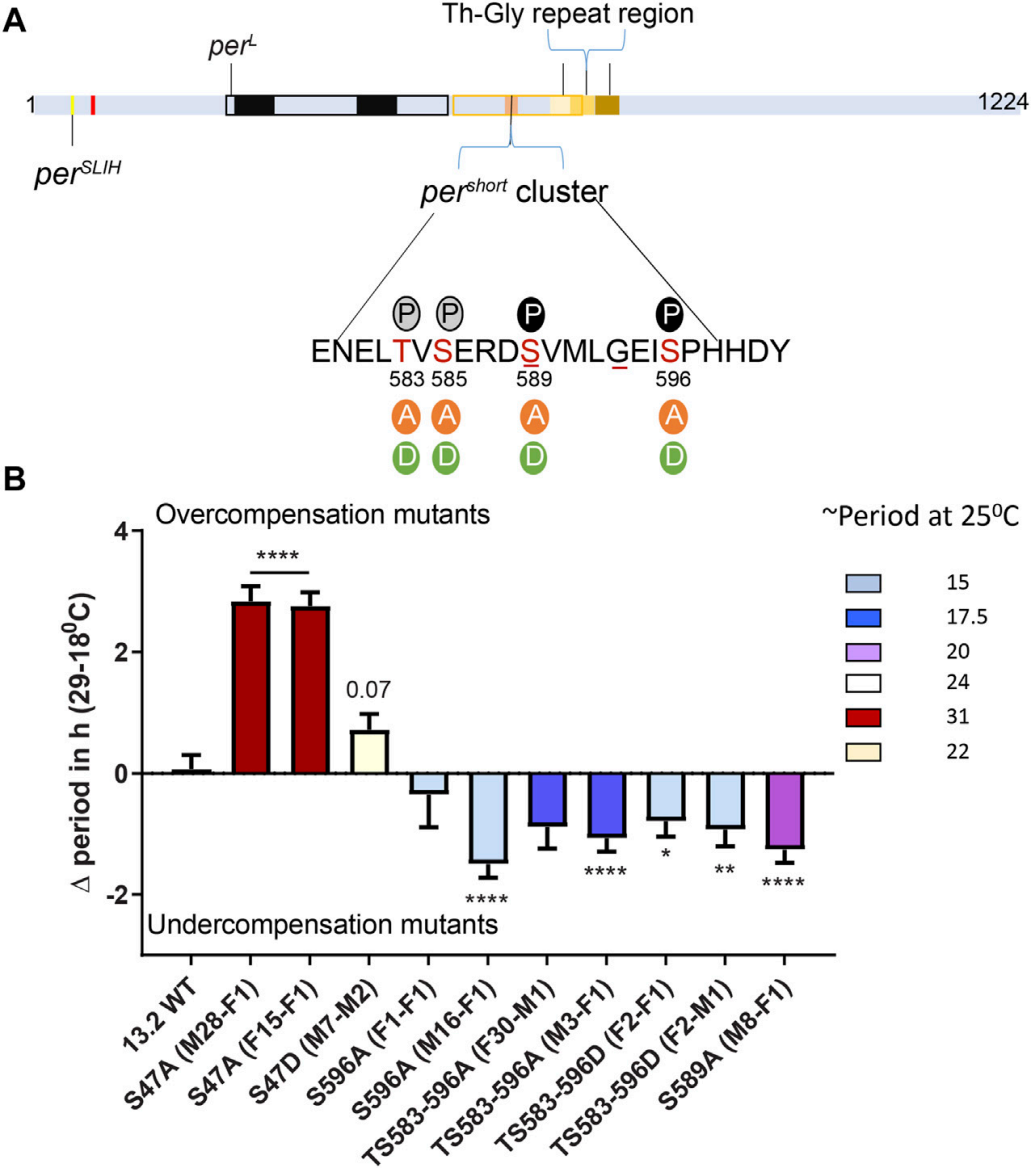
The *per* ^*s*^ phosphocluster impacts temperature compensation. **(A)** PER schematic depicting the relative position of various mutants affecting temperature compensation, as shown in Figure 1A. The magnified region shows key Serine residues of the *per* ^*s*^ phosphocluster and the various substitutions that were tested. Solid P within black circle at 589 and 596 shows sites of DBT and NMO mediated phosphorylation, respectively. Grey circles show predicted phosphorylation sites at S585 and S583. Underlines at S589 and G593 denote positions of *per* ^*s*^ and *per* ^*T*^ mutant, respectively. **(B)** Graphical representation of the difference in period of various mutants of the *per* ^*s*^ domain at 29 and 18°C. The color scheme denotes period of each mutant line at 25°C. All mutants in the *per* ^*s*^ domain produce short period phenotype at 25°C. Most show significant undercompensation. Transgenic lines with S47A recapitulated our observations with CRISPR-Cas9 genome editing (Figure 1). Transgenic S47D showed a weaker phenotype than genome edited S47D, just short of statistical significance (*p* = 0.07). Statistical significance was calculated using two-way ANOVA with Sidak’s multiple comparison test.

We used previously described transgenic lines (Chiu et al., 2011), in which the *per* ^*0*^ mutation is rescued with *per* transgenes carrying various mutations in the *per* ^*s*^ domain. In addition, we included transgenic flies carrying S47A and S47D substitutions (Chiu et al., 2008). Strikingly, the period length and overcompensation phenotypes observed with transgenes encoding PER S47A were similar to those observed with the corresponding mutants generated with CRISPR-CAS9 (Figures 1, 2), confirming the critical role played by the S47 residue in temperature compensation. For S47D, effect on temperature compensation was weaker with a single mutant transgene than with the CRISPR-Cas9 generated mutant line, and was just above the significance cut-off. Mutants in the *per* ^*s*^ domain exhibited shortened period length at 25°C [as described previously (Chiu et al., 2011)]. They also showed consistently an undercompensation phenotype of about 1–1.5 h, except for one S596A line (Figure 2B). This could be because of an insertional effect of P-elements, since *per* transgenes were randomly inserted in the fly genome. Positional effect of transgene insertions might also explain the weaker phenotype observed with the S47D transgene. It should be noted that the *per* ^*T*^ (G593D substitution) and *per* ^*s*^ (S589N substitution) mutations have also been reported to be undercompensated (Konopka et al., 1994; Rothenfluh et al., 2000; Bao et al., 2001), and that in a recent study similar results as ours have been obtained with the same transgenic lines (Giesecke et al., 2021). We would also like to point out that TS583-596A and TS583-596D mutations both showed an undercompensation phenotype in line with their short period phenotype. Short period length of TS583-596D mutants was also observed previously (Chiu et al., 2011). As discussed above, the inability of Aspartate to fully mimic a dynamic phosphorylation event might explain the similar phenotypes observed with S to A and S to D substitutions.

Taken together, our results and the aforementioned previous and recent reports show that the *per* ^*s*^ domain is important for temperature compensation. Since S to A/D substitutions in this domain have opposite effects than S47A, and since mutagenesis studies revealed that phosphorylation of the *per* ^*s*^ domain blocks S47 phosphorylation (Chiu et al., 2011), it seems likely that the *per* ^*s*^ domain controls temperature compensation through S47 phosphorylation.

### S47A Mutants Show Increased Hyper-Phosphorylated PER Accumulation at Warmer Temperatures

**FIGURE 3:**
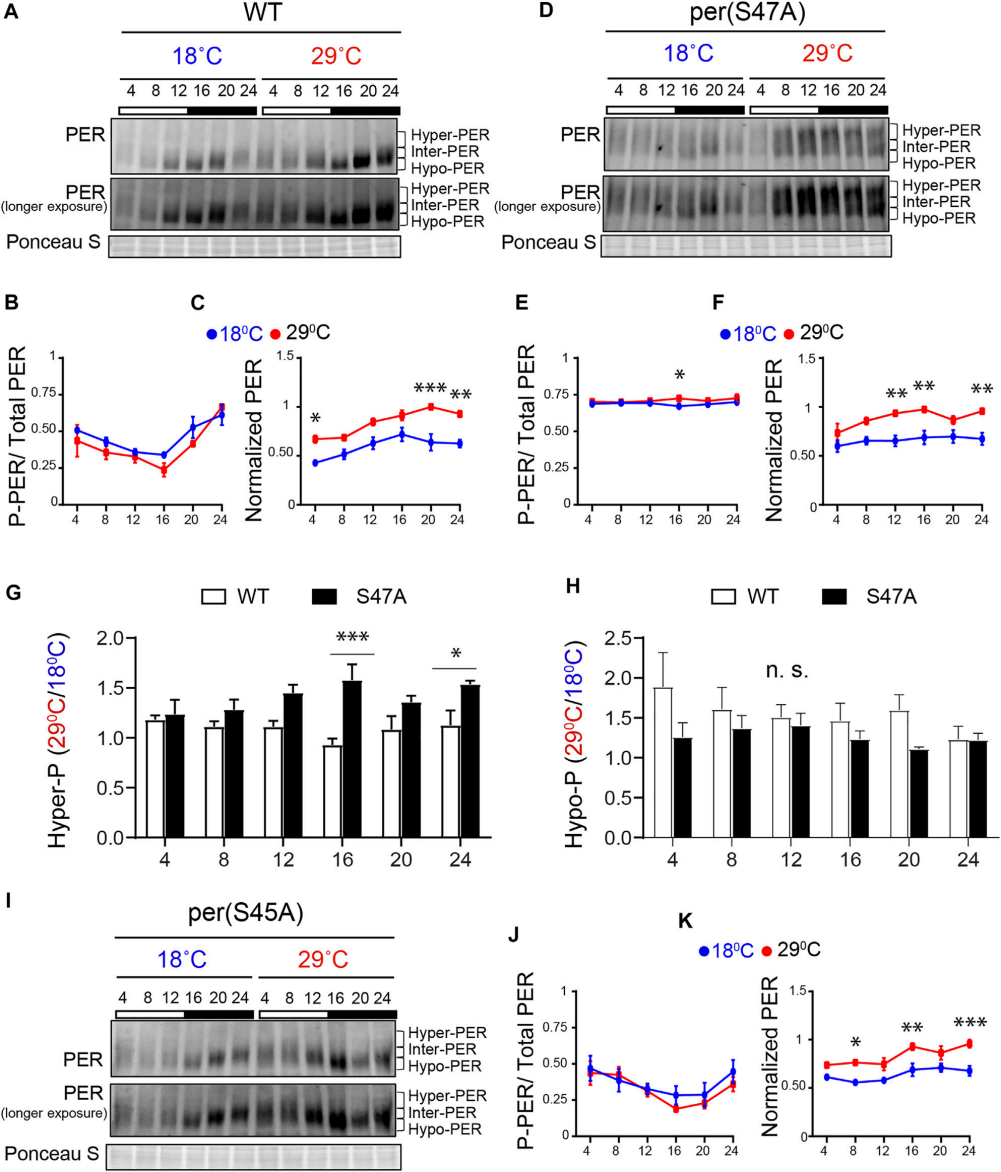
S47A mutation causes hyper-phosphorylated PER to accumulate preferentially at high temperature. **(A,D,I)** Representative western blots probing head extracts from flies entrained to LD at 18 and 29°C. PER abundance was monitored throughout the day at the indicated time points. Respective genotypes are represented at the top of each gel. Brackets indicate hyper-, intermediate- (inter-) and hypo-phosphorylated PER isoforms. Two different exposures of the same blot are shown. Ponceau S was used as a loading control and for normalization. **(B,F)** and **(J,K)** Graphs show PER abundance and phosphorylation (inter + hyper) quantifications. In control flies, both PER phosphorylation **(B)** and abundance **(C)** were scored as statistically rhythmic at 29 and 18°C, using the JTK_CYCLE. For phosphorylation: *p* < 0.05, phase = 2 at 18°C and *p* < 0.001, phase = 2 at 29°C. For abundance: *p* < 0.05, phase = 18 at 18°C and *p* < 0.001, phase = 20 at 29°C. For S47A, neither phosphorylation **(E)** nor abundance **(F)** were scored as statistically rhythmic, although PER levels were reproducibly lower at ZT4. For S45A **(J,K)**, only PER abundance at 18°C scored as statistically rhythmic. *p* < 0.05, phase = 18. Red lines represent data at 29°C and blue lines represent data at 18°C. *p < 0.05, **p < 0.01, ***p < 0.001, two-way ANOVA followed by Sidak’s multiple comparison to test for time and temperature-dependent differences (*n* = 3 biological replicates). **(G,H)** Ratio of PER levels measured at 29°C vs. 18°C. G shows the ratio for hyper-phosphorylated PER, and H for hypo-phosphorylated PER. S47A accumulates more hyper-phosphorylated PER during the night than control flies at warm temperatures.

The results just described provide strong evidence for a key role of the PER phosphodegron in temperature compensation. To gain further mechanistic insights, we examined PER protein and phosphorylation cycling in two phosphodegron mutants that showed opposite temperature-compensation phenotypes: S45A and S47A. We examined PER rhythms in these mutants at 18°C and 29°C in head extracts. Note that for phosphorylation quantifications, we divided PER signal in three categories, as previously done for CLK (Kim and Edery, 2006; Yu et al., 2006): hypo-phosphorylated (hypo-PER), intermediate (inter-PER) and hyperphosphorylated (hyper-PER) (Figures 3A,D,I). Overall PER phosphorylation rhythms are shown in Figures 3B,E,J, where phosphorylated PER (P-PER) include both inter- and hyper-PER isoforms. We also quantified and compared hyper-PER and hypo-PER signals in Figures 3G,H to determine whether the PER S47A substitution had a specific effect on hyper-phosphorylated PER degradation, given the key role played by the phosphodegron in degrading hyperphosphorylated PER (Chiu et al., 2008), and the functional relevance of hyper-PER on transcriptional repression (Nawathean and Rosbash, 2004; Kivimäe et al., 2008).

Under LD, control (*w* ^*1118*^) flies showed rhythmic PER abundance and phosphorylation regardless of the temperature, albeit with significantly higher abundance at 29°C (Figures 3A–C). This observation is in line with increased TIM abundance at warm temperature (Kidd et al., 2015). S47A also showed increased PER abundance at 29°C compared to 18°C, but PER abundance and phosphorylation rhythms were perturbed at both temperature (Figures 3D–F). Importantly, S47A mutants showed exaggerated temperature-sensitive accumulation of hyper-phosphorylated PER compared to controls, which appeared most pronounced at night (see ZT16 and ZT24 in Figure 3G). This was not the case for hypo-phosphorylated PER, which tend to be lower (Figure 3H). Increased accumulation of hyper-phosphorylated PER is likely to result in longer CLK/CYC repression phase, thus lengthening circadian period (Kivimäe et al., 2008) at warmer temperatures. These results therefore reveal a temperature-dependent effect on PER phosphorylation and/or degradation of the S47A substitution, which could underlie the observed overcompensation phenotype.

Though S45A showed a long period and an undercompensation behavior, the effect of S45A mutation on PER cycling was milder than S47A, with patterns of phosphorylation similar to that of WT (Figures 3I–K). However, PER cycling amplitude at 29°C was reduced and did not clear statistical significance. Phosphorylation rhythm amplitude of S45A was reduced at both temperatures as well.

### PER Degradation is Overcompensated as a Result of the S47A Substitution

**FIGURE 4:**
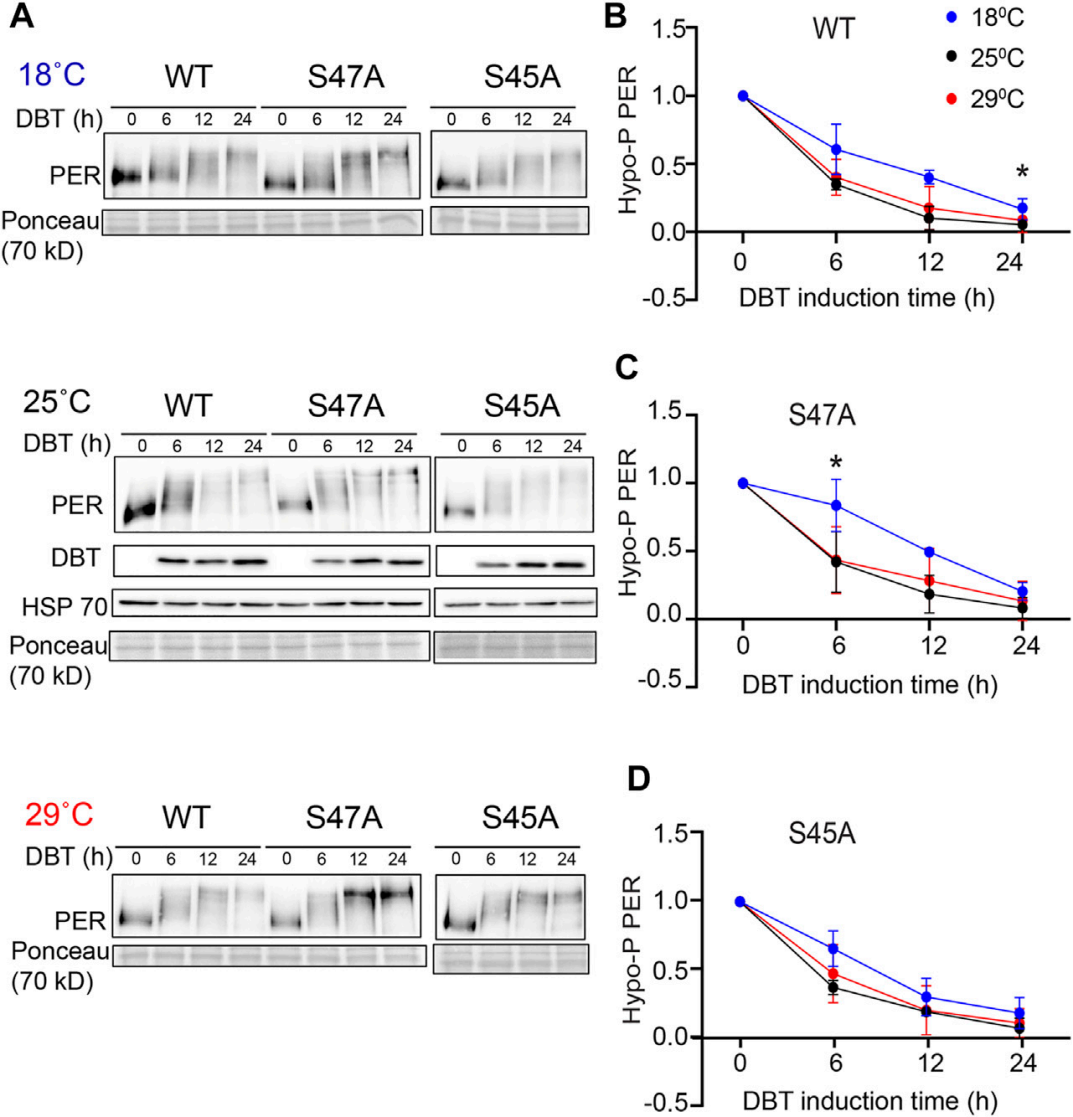
S47A phosphorylation kinetics is slowed down at cold temperature. **(A)** Representative western blots probing cell extracts from S2 cells expressing DBT and either wild-type (WT) PER, S47A or S45A. Cells were incubated at the indicated temperatures, and collected at the indicated time points after DBT induction. Top panel shows PER immunoblotting, bottom panel shows Ponceau S staining, used as loading control and for normalization. **(B–D)** Western Blot quantifications. PER signal shown at time point 0 is used as a reference to classify PER isoforms as hypo-phosphorylated. Graphs representing the relative amount of hypo-phosphorylated PER at 18, 25, and 29°C for the respective genotypes at different time points after DBT induction. S47A shows increased hypo-phosphorylated PER 6 h after DBT induction.*p < 0.05, 18 compared to 29°C, two-way ANOVA followed by Sidak’s multiple comparison to test for time and temperature-dependent differences (*n* = 3 biological replicates).

The increase in the accumulation of hyper-phosphorylated PER, observed at high temperature with the S47A substitution, could be the result of abnormal protein stability, possibly due to disrupted phosphorylation. We therefore turned to S2 cell culture assays (Ko et al., 2002) to determine the effect of temperature on PER phosphorylation and degradation kinetics. In these assays, recombinant DBT is expressed under the inducible metallothionine promoter, while PER is expressed constitutively. Since PER is phosphorylated on multiple sites by DBT and thus produces several phosphorylated isoforms with various mobility that are subjected to SLIMB-mediated proteasomal degradation, we quantified the disappearance of the hypo-phosphorylated PER after DBT induction to determine PER phosphorylation kinetics (Figure 4). Interestingly, phosphorylation kinetics of PER S47A was significantly slowed down at low temperature 6 h after DBT induction (Figures 4A,C), while no significant effect was observed with either wild-type PER or S45A at this time point (Figures 4A,B,D). However, it seems unlikely that a slower phosphorylation kinetic at cold temperature would result in the shorter circadian behavior period observed in S47A mutants at 18°C compared to 29°C.

**FIGURE 5:**
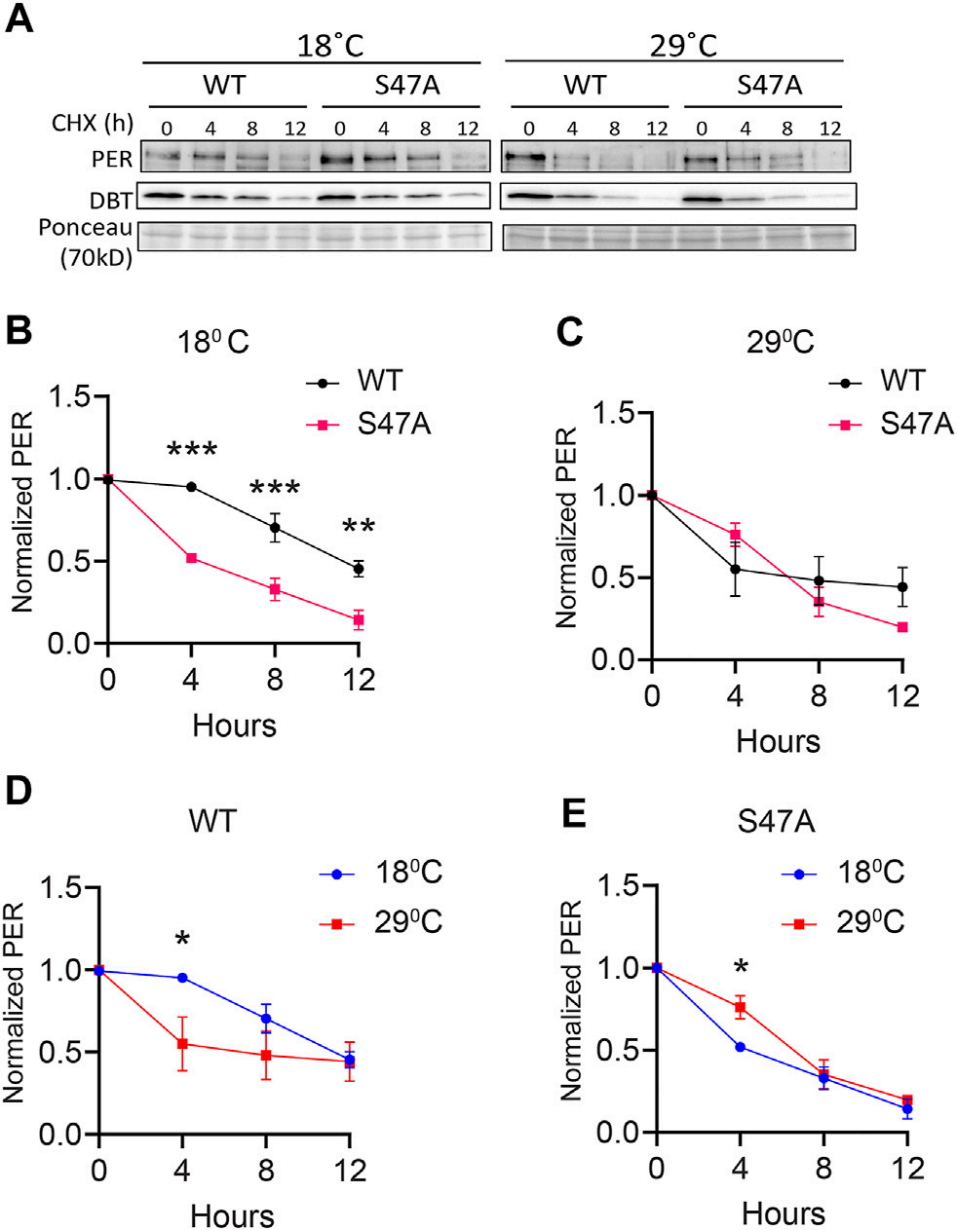
S47A degradation kinetics is excessively temperature-compensated. **(A)** Representative western blots probing cell extracts from S2 cells expressing DBT and either wild-type (WT) PER, S47A or S45A. Cells were collected at the indicated time points after cycloheximide addition at the indicated temperatures. Top panel shows PER immunoblotting, bottom panel shows Ponceau S staining, used as loading control and for normalization. **(B–E)** Western Blot quantifications. All experiments were performed with three independent replicates. *p < 0.05, **p < 0.01, ***p < 0.001, two-way ANOVA followed by Sidak’s multiple comparison to test for time and temperature-dependent differences. In **(B,C)**, WT and S47A mutants are compared to each other at the indicated temperature. **(D,E)** show the same data, but degradation kinetics of WT and S47A are compared between high and low temperatures.

**FIGURE 6:**
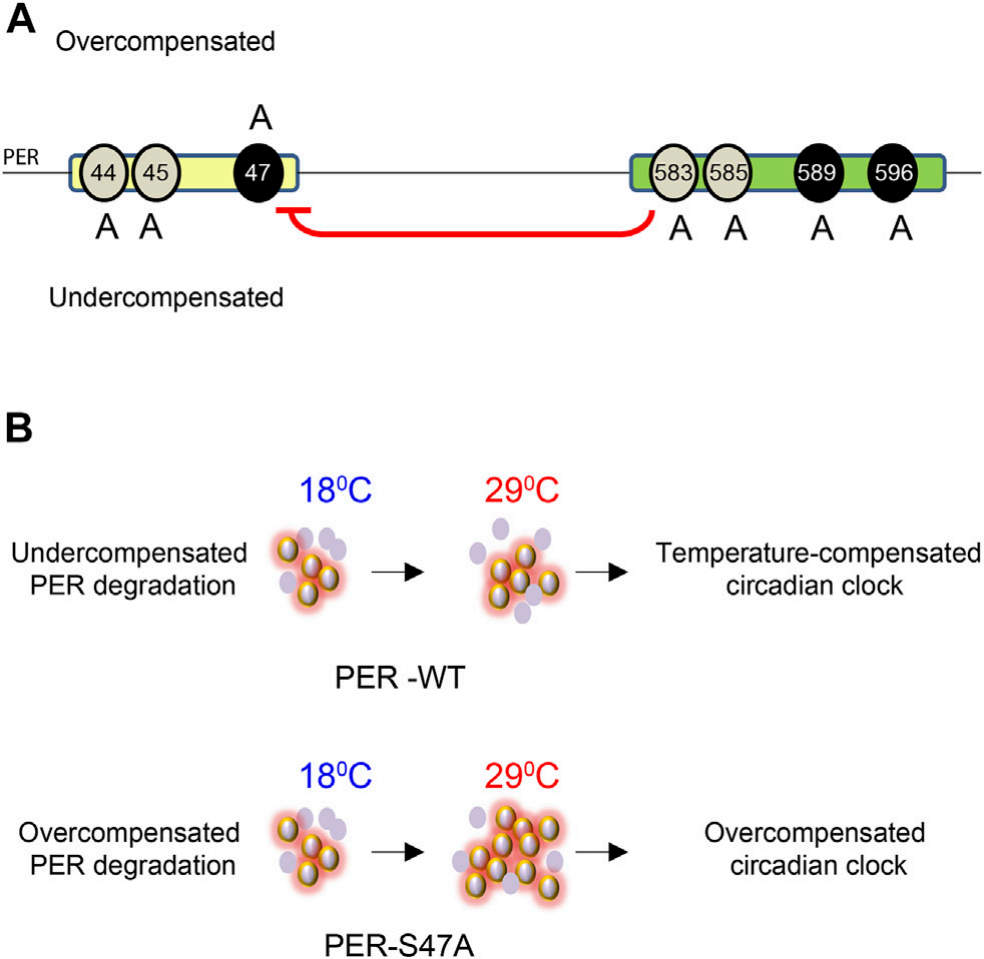
Model for the role of PER phosphoclusters in temperature compensation. **(A)** The PER phosphodegron (yellow) and *per* ^*s*^ phosphocluster (green) modulate temperature compensation. Mutations of different residues of the PER phosphodegron have opposite effects on temperature compensation. S44A and S45A cause undercompensation while S47A results in overcompensation of circadian period. Mutations in the *per* ^*s*^ phosphocluster cause undercompensation, consistent with its inhibitory role on S47 phosphorylation (red arrow). Black ovals around 47, 589, and 596 show DBT and NMO mediated phosphorylation sites on PER. Grey ovals around 44, 45, 583, and 585 depict putative additional phosphorylation sites. **(B)** S47 action on temperature compensation. In wild-type flies, PER abundance and rate of PER degradation increase with increase in temperature. Hyper- and hypo-phosphorylated isoforms remain in balance and the clock is properly temperature-compensated, presumably because of S47 phosphorylation. In S47A mutant flies, PER degradation is overcompensated and thus slows down at high temperature, leading to excessive accumulation of hyperphosphorylated isoforms. This is predicted to prolong repression of CLK/CYC-transcription and thus lengthened period (Kivimäe et al., 2008). The clock is then overcompensated. Violet ovals represent PER protein, Halo around violet ovals show hyper-phosphorylated PER.

Since S47A caused increased hyper-phosphorylated PER accumulation at 29°C, we also determined S47A’s effects on PER stability. We again induced DBT and after 6 h added cycloheximide to block protein synthesis. To quantify the amount of PER protein more accurately, we deliberately condensed different phosphorylated PER isoforms in this assay by running a higher percentage SDS-PAGE gel. Wild-type PER was more stable than S47A at 18°C, while at 29°C there was no obvious difference (Figures 5B,C). Interestingly, S47A’s degradation kinetics was slowed down at higher temperature, while for WT, it was accelerated (Figures 5A,D,E). Thus, S47A results in a slower degradation of PER at high temperatures, which we propose would contribute to the excessive hyper-phosphorylated PER visible in flies expressing S47A, and thus would cause overcompensation of circadian period (Figure 6).

## Discussion

Phosphorylation events play an important role in the temperature compensation of the circadian clock across phyla (Tosini and Menaker, 1998; Mehra et al., 2009; Portolé and Má, 2010; Zhou et al., 2015; Masuda et al., 2020). In *Drosophila*, PER phosphorylation has been studied in detail, and interestingly three *per* mutations affecting two PER domains undergoing DBT phosphorylation are undercompensated. *per* ^*SLIH*^ causes the most robust undercompensation phenotype (Hamblen et al., 1998), while *per* ^*s*^ and *per* ^*T*^ have milder effects (Ewer et al., 1990; Konopka et al., 1994; Rothenfluh et al., 2000; Bao et al., 2001). We therefore systematically tested phosphorylated residues in both the PER phosphodegron and the *per* ^*s*^ domain to understand their impact on temperature compensation. Overall, our results point at a central role for residue S47 through temperature-dependent modulation of PER degradation, and a differential, yet to be elucidated, role of S44 and S45. In addition we found that phosphorylated residues in the *per* ^*s*^ phosphorylation cluster also play a role in temperature compensation, as expected from their ability to inhibit S47 phosphorylation (Chiu et al., 2011).

Indeed, the S47A substitution resulted in a striking overcompensation. This suggests that phosphorylation at this residue is important for the compensation. Actually, we found that PER levels were exaggeratedly temperature-dependent *in vivo*, with an increase in hyper-phosphorylated PER levels at high temperature. Moreover, PER degradation induced by DBT phosphorylation was slowed down at high temperature by the S47A substitution, as opposed to WT PER in a *Drosophila* cell culture assay. These results thus suggest that the S47 residue impacts the kinetics of PER degradation in a temperature-dependent manner, protecting degradation from being overcompensated. It should be noted that our molecular observations were made with whole-head extracts (and S2 cells), but rhythmic locomotor behavior is controlled by a small number of circadian pacemaker neurons, the small ventral lateral neurons (sLNvs), that contribute minimally to whole-head protein extracts (Zeng et al., 1994; Renn et al., 1999; Stoleru et al., 2005). Differences have been noted between the molecular clock of these neurons and other circadian neurons and tissues (Lin et al., 2002; Lim et al., 2011; Top et al., 2016; Reviewed in; Top and Young, 2018). We therefore cannot exclude that S47A impacts differently PER rhythms in sLNvs than in other tissues, but we note importantly that DBT-mediated PER phosphorylation and degradation is mechanistically conserved in clock neurons and peripheral tissues, and closely recapitulated in S2 cells (Kloss et al., 1998, 2001; Price et al., 1998; Ko et al., 2002; Chiu et al., 2008, 2011; Garbe et al., 2013).

Surprisingly, our results from cycloheximide chase assays suggest that S47A is less stable than WT at 18°C, while at 29°C we observed a mild increase in stability of S47A compared to WT. These results appear to differ from those reported in Chiu et al. (2008) that indicated a robust stabilization of S47A at 25°C. These discrepancies could be the result of slightly different growth conditions or other changes in the state of our S2 cell cultures, which could affect expression of endogenous proteins important for PER degradation. Nevertheless, since the increase in stability of the S47A protein at 29°C correlates well with the overcompensation of circadian period in S47A flies, our observations in S2 cells provide a coherent mechanistic explanation for S47A’s temperature-compensation phenotype. However, that circadian behavior period is lengthened at all temperatures in S47A mutant flies seems at odd with the kinetics of S47A degradation observed in cell culture, which is accelerated at 18°C or similar to wild-type PER at 29°C (Figures 5B,C). The cycloheximide chase assay we used models DBT-dependent PER degradation in the absence of any feedback such as PER repression of its own transcription. The S47A mutation could thus have a lengthening effect on period independent of its impact on temperature compensation, for example by modulating the ability of PER to function as a transcriptional repressor *in vivo*. A different mechanism for period determination and temperature compensation by the phosphodegron is in agreement with our observation that S to A substitutions at residues 44, 45 or 47 all cause period lengthening, while the valence of the temperature compensation phenotype is dependent on the specific residue being substituted.

Mutations in the *per* ^*s*^ phosphocluster provide further support to the key role played by phosphorylation at S47 in temperature compensation. Indeed, mutations in the *per* ^*s*^ phosphocluster, which inhibit S47 phosphorylation, have opposite effects on temperature compensation than S47A: flies carrying these mutations are undercompensated. Unexpectedly though, the phosphomimetic substitution S47D did not produce an undercompensation phenotype. Rather, an overcompensation phenotype was observed. This could be explained by the substitution to Aspartate not fully mimicking the structure of a phosphorylated Serine, or that constantly mimicking S47 phosphorylation impacts thermal compensation of the clock through a different mechanism than the S47A substitution. Notably it is not uncommon that phosphomimetic mutations do not produce opposite phenotype than phosphoinhibitory mutations, including in circadian proteins (Lin et al., 2005; Chiu et al., 2011; Top et al., 2018; Cai et al., 2021). Thus, S47D could lead to overcompensation *via* yet unknown, different mechanism. Given the opposite effect of S47A and S47D on SLIMB binding (Chiu et al., 2008) and consequently period length, PER-SLIMB interaction and thus PER degradation is unlikely to be the mechanism mediating S47D temperature overcompensation.

Another unexpected observation is that Serine to Alanine substitution at residues 44 and 45 have opposite effects on temperature compensation than S47A, even though all these substitutions lengthen circadian period. Residues of the phosphodegron are thus functionally heterogeneous when it comes to temperature compensation. Indeed, unlike S47A, S45A did not cause obvious change in temperature-dependent PER stability *in vivo* or in cell culture. It should be noted that unlike S47, S44 or S45 phosphorylation has not been unambiguously demonstrated (Chiu et al., 2008). It remains possible that these residues are not phosphorylated and play a structural role in the phosphodegron, and this could explain functional diversity in temperature compensation between S44/45 and S47. It is also possible that S44/45’s mechanism of action on period length and/or temperature compensation is independent of S47. Clearly, further studies are needed to elucidate S44 and S45’s exact function in thermal compensation. Of note, the S45F substitution, which introduces a more bulky amino acid than S45A, caused a more severe phenotype that better mimicked the *per* ^*SLIH*^ phenotype resulting from a S45Y substitution [Figure 1B; Table 1 (Hamblen et al., 1998)]. This could indicate an important structural role for this amino acid, perhaps controlling specific protein-protein interactions not implicated in PER degradation. We note that a putative Nuclear Localization Signal (NLS) is located close to the phosphodegron (Figure 1A, red mark) and perhaps a bulky amino acid at S45 can interfere in a temperature dependent manner with nuclear translocation. However, at least in S2 cells, this NLS has little impact on nuclear localization (Chang and Reppert, 2003).

Interestingly, our work, combined with previous work in mammals, converge to support the model that CKIδ/ε-mediated phosphorylation of PER proteins and their proteasomal degradation are critical for temperature compensation of the circadian clock. Surprisingly, however, there appear to be important mechanistic differences. Indeed, in *Drosophila*, mutation of the key Serine residue (S47) in the phosphodegron increases temperature compensation, while in mammals mutation of the homologous S478 residue has the opposite effect [Figures 1B, 2B; Table 1 (Zhou et al., 2015; Masuda et al., 2020)]. Intriguingly, in mammalian cell culture assays, phosphodegron mutation (S478A in mammals) causes degradation kinetics to become essentially insensitive to temperature (Zhou et al., 2015), while S47A degradation in flies is mildly overcompensated. However, in mammals, wild-type mPER2 degradation kinetics accelerates when temperature drops, while in flies we observed the opposite effect, perhaps because the phosphodegrons, though conserved, are located in different location in the PER and mPER2 proteins. This explains why mutations that both renders PER degradation less sensitive to temperature have opposite effect on temperature compensation.

Finally, it is important to point out that so far, any mutation or manipulation of phosphorylation of key circadian protein in any system only partially disrupts temperature compensation, or even increases it. It is therefore clear that multiple mechanisms are implicated, and recent results by Giesecke and collaborators (Giesecke et al., 2021) in flies for example point to an important role for nuclear export. It is therefore clear that temperature compensation is proving to be a complex, systemic process, as first proposed by Hastings and Sweeny in their seminal work on Dinoflagellates (Hastings and Sweeney, 1957).

## Materials and Methods

### Fly Stocks

Fly stocks were maintained on standard cornmeal agar at 25°C under 12:12 h light: dark (LD). The following available strains were used *w* ^*1118*^, *yw*, *FM7a*, *w[1118]; PBac[y(+mDint2) = vas-Cas9]VK00027* (BL51324), *y[1] M(Act5C-Cas9.P.RFP-)ZH-2A w[1118] DNAlig4*[169] (BL58492). Transgenic flies used in Figure 2 were described previously (Chiu et al., 2008, Chiu et al., 2011).

### CRISPR Mutagenesis

Single stranded oligos (ssODNs, Table 2) were injected into either BL51324 or BL58492 from Bloomington. The injected embryos, upon eclosion, were crossed with *FM7a* balancer flies. F1 progeny was screened with PCR and sequencing for the presence of the desired modifications. All the resultant lines were backcrossed into the *w* ^*1118*^ genetic background. For lines generated from BL58492 (S45S# I, S45S# II, S45D #122, #67, S45A 98, S4445A# 105, S45E, S45F #51, S44D, and S47D), we determined whether the Cas9 transgene located on the X chromosome had been removed. S45E, S44D, and S47D fly lines unexpectedly retained Cas9 even after multiple attempts to remove it. It seems however highly unlikely that Cas9 could somehow interfere with temperature compensation, particularly since in both S45E and S44D temperature compensation was unaffected (Figure 1B). Primers used for molecular screenings are listed in Table 3.

**TABLE 2:** ssODNs for *per* mutagenesis.

**Genotype**	**ssODN sequence in 5′ to 3′ direction**
S45S	ATC​CAT​CCC​CTT​TCC​AGT​GGC​AGC​TCC​AAA​TCC​CGC​CTG​AGC​GGC​AGTCAC​TCC​TCC​GGC​AGC​AGT​GGC​TAT​GGG​GGC​AAG​CCC​TCG​ACG​CAG​GCC​AGC​AGC​AGC​GAC​AT
S45A	ATC​CAT​CCC​CTT​TCC​AGT​GGC​AGC​TCC​AAA​TCC​CGC​CTG​AGC​GGC​AGT​CAC​TCC​GCG​GGC​AGC​AGT​GGC​TAT​GGG​GGC​AAG​CCC​TCG​ACG​CAG​GCC​AGC​AGC​AGC​GAC​AT
S45D	ATC​CAT​CCC​CTT​TCC​AGT​GGC​AGC​TCC​AAA​TCC​CGC​CTG​AGC​GGC​AGT​CAC​TCC​GAC​GGC​AGC​AGT​GGC​TAT​GGG​GGC​AAG​CCC​TCG​ACG​CAG​GCC​AGC​AGC​A\GCG​ACA​T
S45F	ATC​CAT​CCC​CTT​TCC​AGT​GGC​AGC​TCC​AAA​TCC​CGC​CTG​AGC​GGC​AGT​CAC​TCC​TTC​GGC​AGC​AGT​GGC​TAT​GGG​GGC​AAG​CCC​TCG​ACG​CAG​GCC​AGC​AGC​AGC​GAC​AT
S45E	ATC​CAT​CCC​CTT​TCC​AGT​GGC​AGC​TCC​AAA​TCC​CGC​CTG​AGC​GGC​AGT​CAC​TCC​GAG​GGC​AGC​AGT​GGC​TAT​GGG​GGC​AAG​CCC​TCG​ACG​CAG​GCC​AGC​AGC​AGC​GAC​AT
S44A	ATC​CAT​CCC​CTT​TCC​AGT​GGC​AGC​TCC​AAA​TCC​CGC​CTG​AGC​GGC​AGT​CAC​GCG​TCC​GGC​AGC​AGT​GGC​TAT​GGG​GGC​AAG​CCC​TCG​ACG​CAG​GCC​AGC​AGC​AGC​GAC​AT
S44D	ATC​CAT​CCC​CTT​TCC​AGT​GGC​AGC​TCC​AAA​TCC​CGC​CTG​AGC​GGC​AGT​CAC​GAC​TCC​GGC​AGC​AGT​GGC​TAT​GGG​GGC​AAG​CCC​TCG​ACG​CAG​GCC​AGC​AGC​AGC​GAC​AT
S47A	ATC​CAT​CCC​CTT​TCC​AGT​GGC​AGC​TCC​AAA​TCC​CGC​CTG​AGC​GGC​AGT​CAC​TCC​TCC​GGC​GCG​AGT​GGC​TAT​GGG​GGC​AAG​CCC​TCG​ACG​CAG​GCC​AGC​AGC​AGC​GAC​AT
S47D	ATC​CAT​CCC​CTT​TCC​AGT​GGC​AGC​TCC​AAA​TCC​CGC​CTG​AGC​GGC​AGT​CAC​TCC​TCC​GGC​GAC​AGT​GGC​TAT​GGG​GGC​AAG​CCC​TCG​ACG​CAG​GCC​AGC​AGC​AGC​GAC​AT
S44-45A	ATC​CAT​CCC​CTT​TCC​AGT​GGC​AGC​TCC​AAA​TCC​CGC​CTG​AGC​GGC​AGT​CAC​GCG​GCG​GGC​TTC​AGT​GGC​TAT​GGG​GGC​AAG​CCC​TCG​ACG​CAG​GCC​AGC​AGC​AGC​GAC​AT
S45-47A	ATC​CAT​CCC​CTT​TCC​AGT​GGC​AGC​TCC​AAA​TCC​CGC​CTG​AGC​GGC​AGT​CAC​TCC​GCG​GGC​GCG​AGT​GGC​TAT​GGG​GGC​AAG​CCC​TCG​ACG​CAG​GCC​AGC​AGC​AGC​GAC​AT

**TABLE 3:** Molecular screening for *per* mutagenesis.

**Product name**	**Product size**	**Forward primer(5′to 3′)**	**Reverse primer(5′to 3′)**
*per*	1332	GTT​ACC​CCC​ATT​CAA​GGT​CC	CCA​AAA​CGG​GCA​CAG​ATA​CT
Act5-Cas9	771	GAT​AAA​AAT​CTG​CCT​AAC​G	CTT​ATG​CCT​TCC​CAT​TAC​T
Vasa-Cas9Set1	762	GATAAGAACCTGCCCAAC	GCCCATCACTTTCACGA
Vasa-Cas9Set2	761	GCCTACCACGAGAAGTA	AGGATGGGCTTGATGAA
*lig4*	784	GCCAGCACGATCAAGTTC	GGTCTGCCAGCAGATTG

### ssODN Design

A conveniently located PAM site (TGG) was identified using CRISPR fly design. The guide RNA sequence chosen was—5′AGCCACTGCTGCCGGAGGAGTGG. The guide RNAs were cloned into plasmid pCFD3 (Addgene, Cat no: 49410). Silent modifications (AGC-AGT) were introduced into the seed sequence to prevent re-cutting and for subsequent molecular screening.

### Circadian Locomotor Activity Monitoring and Analysis

2–5 days old male flies were loaded into behavior tubes. Flies were entrained at the defined temperature for at least 3 days under 12:12 LD cycle and released into constant darkness (DD). Activity was recorded using the DAM (*Drosophila* Activity Monitoring) system (Trikinetics, Waltham, MA, United States) in 136-LL incubators (Percival). Behavior was analyzed and plotted using FaasX software (courtesy of F. Rouyer, Centre National de la Recherche Scientifique, Gif-sur- Yvette, France). Rhythmicity was defined by following the criteria: power >20, width >1.5 h, using the c2 periodogram analysis. 5 days of DD data were used to determine period. Period and power of all individual flies under various conditions can be found in supplemental file Supplementary Figures S1, S2; Supplementary Table S1 —datasets. xls.

## Statistical Analysis

Whether difference in period at 29 and 18°C were statistically significant was determined by using a two-way ANOVA followed by Sidak’s multiple comparison tests. Since in Figure 1 control flies showed a significant period shortening at warm temperatures compared to cold, we did factor this drift prior to run statistical test by subtracting the average period lengthening observed in S45SI, and S45SII (1.03 h) from the period observed at 18°C for each genotypes.

For graphical representation in Figures 1, 2, mean difference between 29 and 18°C were plotted on the Y axis and Standard Error (SE) of difference were used for error bars. Mean difference and SE of difference were obtained from the Two-way Anova statistics details is Prism. Western blots were analyzed using Two-way ANOVA with Tukey’s multiple comparison test. Rhythms in PER phosphorylation and abundance in Figure 3 were analyzed by JTK-CYCLE as in (Hughes et al., 2010).

### *Drosophila* S2 Cell Culture and Transfection

*Drosophila* S2 cells were maintained at 22°C in Schneider’s *Drosophila* medium [Life Technologies (Carlsbad, CA, United States)] supplemented with 10% Fetal Bovine Serum (FBS) (VWR, Radnor, PA). Plasmids expressing pAc-*per*(WT)-V5, pAc-*per*(S45A)-V5, pAc-*per*(S47A)-V5, and pMT-*dbt*-V5 were described previously (Ko et al., 2002; Chiu et al., 2008). For all cell culture experiments, S2 cells were seeded at 1 × 10^6^ cells/ml in a 6-well plate and transfected using Effectene (Qiagen, Germantown, MD, United States). For mobility shift assay (Figure 4), S2 cells were co-transfected with 0.2 μg of pMT-*dbt*-V5 and 0.8 μg of pAc-*per*(X)-V5, where X is either WT, S45A or S47A. *dbt* expression were induced with 500 μM CuSO_4_ 36 h after transfection and cells were moved into incubators with indicated temperature and cells were harvested at 0, 6, 12, 24 h after induction. Proteins were analyzed by Western Blotting. For cycloheximide (CHX) chase experiment (Figure 5), S2 cells were co-transfected with 0.2 μg of pMT-*dbt*-V5 and 0.8 μg pAc-*per*(X)-V5, where X is either WT or S47A. *dbt* expression were induced with 500 μM CuSO_4_ at 36 h after transfection. 6 h after DBT induction, CHX (Sigma) was added at a final concentration of 10 μg/ml. Cells were harvested and lysed with EB2 (20 mM HEPES pH 7.5, 100 mM KCl, 5% glycerol, 5 mM EDTA, 1 mM DTT, 0.1% Triton X-100, 10 μg/ml Aprotinin, 5 μg/ml Leupeptin, 1 μg/ml Pepstatin A, 0.5 mM PMSF, 25 mM NaF) at the indicated times. Proteins were analyzed by Western Blotting.

### Western Blotting and Antibodies

Protein extractions from *Drosophila* S2 cells and adult fly heads, western blotting, and image analysis was performed as previously described (Chiu et al., 2008; Cai et al., 2021). 2–5 day old flies were entrained to 12:12 light-dark cycle for 3 days at indicated temperature. On the 4th day of LD, flies were collected at the indicated time points. ∼40 fly heads for each time point were extracted with RBS buffer (20 mM HEPES pH7.5, 50mM KCl, 10% glycerol, 2 mM EDTA, 1 mM DTT, 1% Triton X-100, 0.4% NP-40, 10 μg/ml Aprotinin, 5 μg/ml Leupeptin, 1 μg/ml Pepstatin, 0.5 mM PMSF, 25 mM NaF). Protein concentration was measured using Pierce Coomassie Plus Assay Reagents (Thermo Fisher Scientific). 2X SDS sample buffer was added and the mixture boiled at 95°C for 5 min. Equal amounts of proteins were resolved by polyacrylamide-SDS gel electrophoresis (PAGE) and transferred to nitrocellulose membrane (Bio-Rad, Hercules, CA, United States) using Semi-Dry Transfer Cell (Bio-Rad). Membranes were stained with Ponceau S solution (0.1% Ponceau S, 5% acetic acid) to stain total proteins as loading control (Chen et al., 2011). Membranes were then incubated in 5% Blocking Buffer (Bio-Rad) for 40 min, incubated with primary antibodies for 16–20 h. Blots were then washed with 1X TBST for 1 h, incubated with secondary antibodies for 1 h, washed again prior to treatment of chemiluminescence Clarity ECL reagent (Bio-Rad). The following percentage of polyacrylamide-SDS gel were used: 6% for PER (Figures 3, 4); 10% for DBT and HSP70.10% gel was used for PER in order to condense the bands for quantification of total PER abundance in CHX chase experiment (Figure 5). For Figures 3B,E,J phospho-PER (intermediate and hyperphosphorylated moieties were quantified as showed in the brackets in Figures 3A,D,I. For Figures 3G,H, only hyper-phosphorylated form of PER was used for analysis. WT, ZT 16 was used to define hypo-phosphorylated form of PER.

Primary antibodies: α-V5 (Thermo Fisher Scientific) at 1:3000 for PER-V5 and DBT-V5, α-PER (GP5620; RRID:AB_2747405) at 1:2000 for PER, α-HSP70 at 1:10000 for HSP70. Secondary antibodies conjugated with HRP were added as follows: α-mouse IgG (Sigma) at 1:2000 for α-V5 detection, 1:10000 for α-HSP70 detection, α-guinea pig IgG (Sigma) at 1:1000 for α-PER detection. All quantifications are included in supplemental file Supplementary Figures S3-S5 - datasets.xlsx.

## Data Availability

The raw data supporting the conclusion of this article will be made available by the authors, without undue reservation.
